# Supplementary material for: The Association between Dietary Protein Intake and Sources and the Rate of Longitudinal Changes in Brain Structure
Source: Nutrients. 2024 Apr 25;16(9):1284. doi: 10.3390/nu16091284 (PMC11085529; doi:10.3390/nu16091284)
Supplement: Supplementary file 1 [file nutrients-16-01284-s001.zip › nutrients-2950722-supplementary.pdf]

The association between dietary protein intake and sources and the rate of longitudinal changes in brain structure

Fusheng Cui , Huihui Li, Yi Cao, Weijing Wang, Dongfeng Zhang

Supplemental Table S1 Dietary Protein Sources Classification and Codes

Protein sources	ID	items
	26066	Beef
red meat	26100	Lamb
	26117	Pork
Processed meat	26122	Processed meat
Poultry	26121	Poultry
Eggs	26088	Egg and egg dishes
cheese	26099	High fat cheese
	26103	Medium and low-fat cheese
Nuts	26107	Unsalted nuts and seeds
	26108	Salted nuts and seeds
yogurt	26102	Low fat yogurt
	26096	Full fat yogurt
Whole grains	26105	Muesli
	26076	Bran cereal
	26077	Oat cereal (non sugar)
	26078	Oat cereal (sugar)
	26079	Other cereal (sugar)
	26074	Whole meal bread
	26114	Whole meal pasta, brown rice and other wholegrains
Legumes	26086	Soy desserts and yogurt
	26136	Soy milk
	26115	Peas and sweetcorn
	26137	Meat substitutes - soy
	26101	Legumes and pulses
milk	26150	Whole milk
	26131	Semi skimmed milk
	26133	Skimmed milk and cholesterol-lowering milk
sea food	26109	Oily fish
	26149	White fish and tinned tuna
	26070	Breaded/battered fish

The questionnaire was first introduced as part of the Assessment visit towards the end of recruitment for the last 70,000 participants. Participants who had provided UK Biobank with e-mail addresses were also invited, via e-mail, to complete the questionnaire online on four separate occasions between Feb 2011 and April 2012.

Assessment center: April 2009 to September 2010

1 st e-mail invitations: Feb 2011 - April 2011

2 nd email invitations: June 2011 - Aug 2011

3 rd email invitations: Oct 2011 – Dec 2011

4 th email invitations: April 2012 – June 2012

Supplemental Table S2 Baseline characteristics of study participants on white matter hyperintensities(N=2679)

	Total	Female	Male	P-value
N	2679	1386	1293	
age, y (mean (SD))	52.70 (7.41)	51.71 (7.15)	53.77 (7.54)	<0.001
sex (%)				
female	1386 (51.7)	1386 (100.0)	0 (0.0)	
male	1293 (48.3)	0 (0.0)	1293 (100.0)	
MET (%)				
low	466 (17.4)	229 (16.5)	237 (18.3)	0.404
medium	1101 (41.1)	582 (42.0)	519 (40.1)	
high	1112 (41.5)	575 (41.5)	537 (41.5)	
TDI (mean (SD))	-2.00 (2.64)	-1.92 (2.68)	-2.08 (2.59)	0.127
smoke (%)				
never	1719 (64.2)	905 (65.3)	814 (63.0)	0.221
ever smoker	960 (35.8)	481 (34.7)	479 (37.0)	
race (%)				
others	75 (2.8)	41 (3.0)	34 (2.6)	0.691
white	2604 (97.2)	1345 (97.0)	1259 (97.4)	
drink (%)				
never	54 (2.0)	34 (2.5)	20 (1.5)	0.126
ever drinker	2625 (98.0)	1352 (97.5)	1273 (98.5)	
education (%)				
below	1204 (44.9)	605 (43.7)	599 (46.3)	0.176
college or above	1475 (55.1)	781 (56.3)	694 (53.7)	
BMI (%)				
Underweight	16 (0.6)	13 (0.9)	3 (0.2)	<0.001
Normal weight	1124 (42.0)	718 (51.8)	406 (31.4)	
Overweight and obesity	1539 (57.4)	655 (47.3)	884 (68.4)	
cancer (%)	219 (8.2)	133 (9.6)	86 (6.7)	0.007
CVD (%)	73 (2.7)	7 (0.5)	66 (5.1)	<0.001
hypertension (%)	519 (19.4)	160 (11.5)	359 (27.8)	<0.001
DM (%)	79 (2.9)	25 (1.8)	54 (4.2)	<0.001
energy (mean (SD))	8773.90 (2260.05)	8228.82 (2004.52)	9358.19 (2370.56)	<0.001
total protein (mean (SD))	81.75 (22.74)	78.14 (20.17)	85.63 (24.63)	<0.001
animal protein (mean (SD))	53.05 (20.13)	50.81 (18.60)	55.45 (21.41)	<0.001
vegetable protein (mean (SD))	28.71 (9.62)	27.33 (9.03)	30.18 (10.02)	<0.001
proportion of animal protein (mean (SD))	0.64 (0.12)	0.64 (0.12)	0.64 (0.11)	0.74
Proportion of vegetable protein (mean (SD))	0.36 (0.12)	0.36 (0.12)	0.36 (0.11)	0.74
animal/vegetable (mean (SD))	0.26 (0.24)	0.26 (0.25)	0.25 (0.23)	0.725

Data for continuous variables are presented as mean (SD). Data for categorical variables are presented as n (%).

Abbreviations: MET, metabolic equivalent; BMI, body mass index; CVDs, cardiovascular diseases; SD, standard deviation; TDI, Townsend deprivation index; DM, diabetes mellitus

Supplemental Table S3 Association between dietary protein intake and longitudinal change rate of total brain, white matter, grey matter volume(N=2723)

	Total brain volume		white matter volume		grey matter volume	
	β(SE)	P	β(SE)	P	β(SE)	P
total protein						
model1	2.775e-06(1.220e-05)	0.820	-3.584e-06 (2.288e-05)	0.876	7.845e-06 (1.611e-05)	0.626
model2	2.124e-05(1.731e-05)	0.220	6.691e-06 (3.249e-05)	0.837	3.283e-05 (2.29e-05)	0.152
model3	2.115e-05(1.729e-05)	0.221	7.123e-06 (3.245e-05)	0.826	3.218e-05 (2.291e-05)	0.160
animal/protein						
model1	7.233e-04 (2.306e-03)	0.754	-2.733e-03 (4.324e-03)	0.527	3.927e-03 (3.044e-03)	0.197
model2	7.57e-04 (2.332e-03)	0.746	-3.06e-03 (4.376e-03)	0.484	4.249e-03 (3.085e-03)	0.168
model3	6.455e-04 (2.330e-03)	0.782	-3.267e-03 (4.37e-03)	0.455	4.227e-03 (3.085e-03)	0.171
vegetable/protein						
model1	-7.232e-04 (2.306e-03)	0.754	2.733e-03 (4.324e-03)	0.527	-3.927e-03 (3.044e-03)	0.197
model2	-7.57e-04 (2.332e-03)	0.746	3.06e-03 (4.376e-03)	0.484	-4.249e-03 (3.085e-03)	0.168
model3	-6.455e-04 (2.33e-03)	0.782	3.267e-03 (4.37e-03)	0.455	-4.227e-03 (3.085e-03)	0.171
vegetable protein						
model1	-8.835e-06 (2.873e-05)	0.759	-2.383e-07 (5.388e-05)	0.996	-1.779e-05 (3.794e-05)	0.639
model2	1.121e-05 (3.761e-05)	0.766	2.808e-05 (7.057e-05)	0.691	-5.531e-06 (4.976e-05)	0.911
model3	1.364e-05 (3.758e-05)	0.717	3.378e-05 (2.581e-05)	0.792	-6.269e-05 (4.978e-05)	0.900
animal protein						
model1	5.507e-06 (1.370e-05)	0.688	-4.464e-06 (2.568e-05)	0.862	1.393e-05 (1.809e-05)	0.441
model2	1.573e-05 (1.581e-05)	0.32	6.170e-07 (2.967e-05)	0.983	2.836e-05 (2.091e-05)	0.175
model3	1.523e-05 (1.579e-05)	0.335	-2.856e-08 (2.964e-05)	0.999	2.794e-05 (2.092e-05)	0.182
animal/vegetable						
model1	2.835e-04 (1.136e-03)	0.803	-1.294e-03 (2.130e-03)	0.544	1.761e-03 (1.500e-03)	0.240
model2	2.843e-04 (1.149e-03)	0.805	-1.458e-03 (2.156e-03)	0.499	1.894e-03 (1.520e-03)	0.213
model3	2.358e-04 (1.146e-03)	0.84	-1.533e-03 (2.153e-03)	0.477	1.870e-03 (1.52e-03)	0.219

Model 1 was adjusted for age, and sex.

Model 2 was based on model 1 and additionally adjusted for Townsend Deprivation Index, total energy intake, education level, physical activity, smoking, alcohol intake, race, body weight status.

Model 3 was based on model 2 and further adjusted for baseline cancer, CVDs, hypertension, and diabetes

Supplemental Table S4 Association between dietary protein intake and longitudinal change rate of white matter hyperintensities(N=2679)

	white matter hyperintensities	
	β(SE)	P
total protein		
model1	1.644e-04 (2.247e-04)	0.464
model2	9.480e-05(3.183e-04)	0.766
model3	9.494e-05 (3.186e-04)	0.766
animal/protein		
model1	9.985e-03(4.235e-02)	0.814
model2	1.788e-02(4.286e-02)	0.677
model3	1.827e-02 (4.289e-02)	0.670
vegetable/protein		
model1	-9.984e-03 (4.235e-02)	0.814
model2	-1.788e-02(4.286e-02)	0.677
model3	-1.827e-02 (4.289e-02)	0.670
vegetable protein		
model1	-3.084e-04 (5.294e-04)	0.560
model2	3.275e-04(6.934e-04)	0.637
model3	3.339e-04(6.940e-04)	0.631
animal protein		
model1	-1.369e-04 (2.519e-04)	0.587
model2	2.149e-05(2.907e-04)	0.941
model3	2.051e-05(2.908e-04)	0.944
animal/vegetable		
model1	-5.569e-03 (2.084e-02)	0.789
model2	-9.557e-03(2.110e-02)	0.651
model3	-9.910e-03(2.111e-02)	0.639

Model 1 was adjusted for age, and sex.

Model 2 was based on model 1 and additionally adjusted for Townsend Deprivation Index, total energy intake, education level, physical activity, smoking, alcohol intake, race, body weight status.

Model 3 was based on model 2 and further adjusted for baseline cancer, CVDs, hypertension, and diabetes.

Supplemental Figure S1 Non-linear associations of dietary protein with the longitudinal change rate of brain structure markers Using a Restricted Cubic Spline Regression Model (N= 2723)

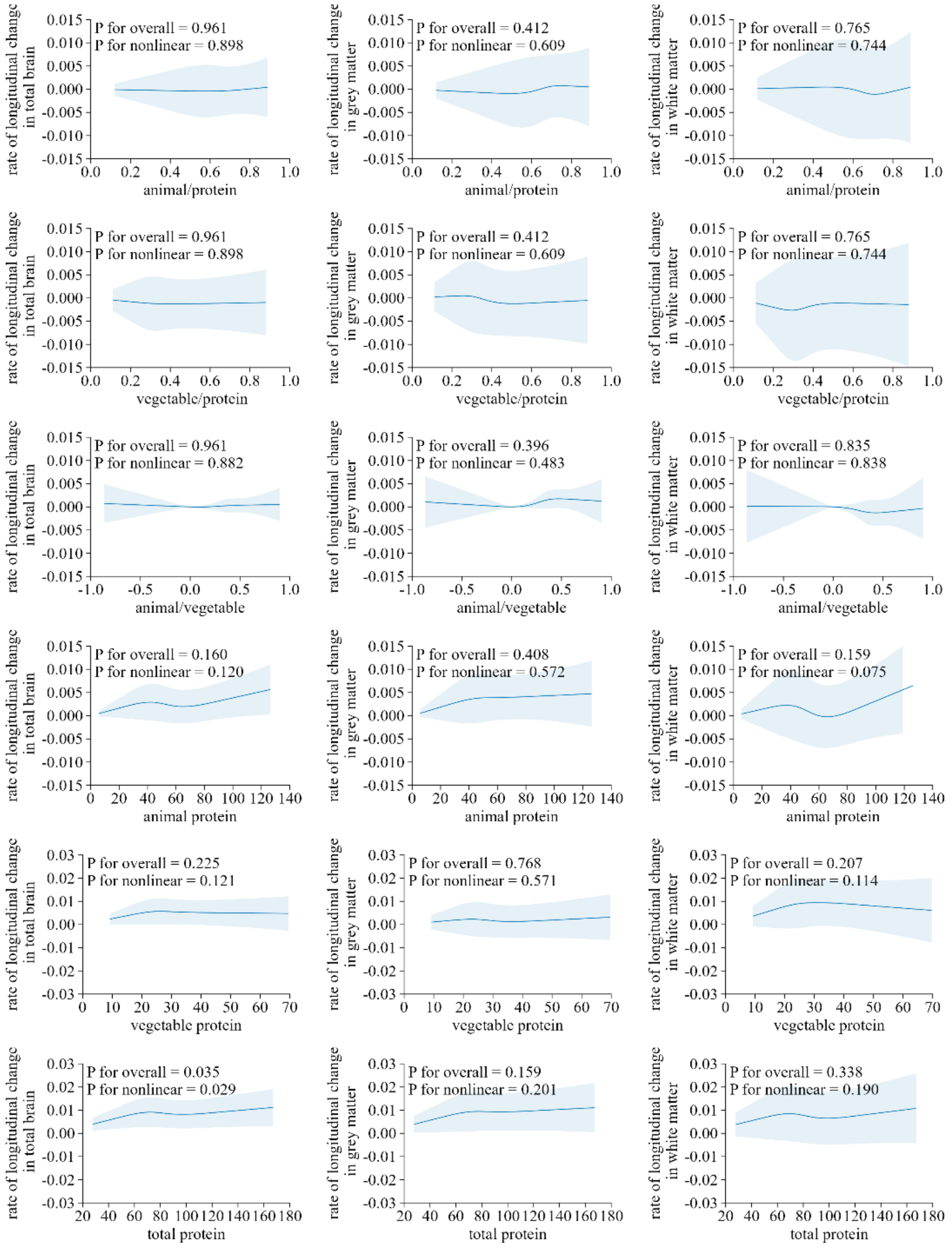

Note: The model was adjusted for age, sex, ethnicity, Townsend deprivation index, education level, physical activity, smoking, body weight status, total energy intake, baseline cancer, CVDs, hypertension, and diabetes.

Supplemental Figure S2 Non-linear associations of dietary protein with the longitudinal change rate of white matter hyperintensities Using a Restricted Cubic Spline Regression Model (N= 2679)

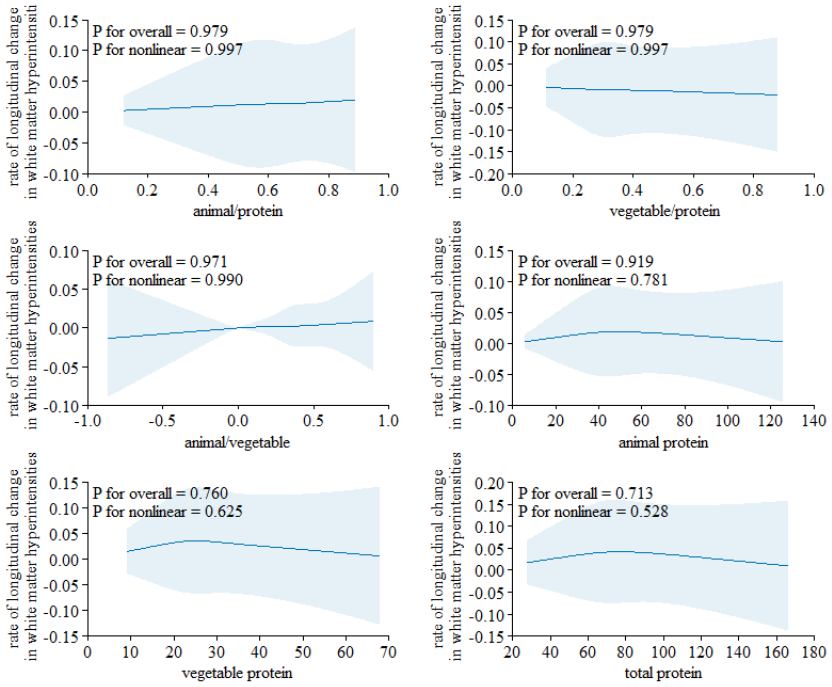

Note: The model was adjusted for age, sex, ethnicity, Townsend deprivation index, education level, physical activity, smoking, body weight status, total energy intake, baseline cancer, CVDs, hypertension, and diabetes.

Supplemental Table S5 Association between dietary protein sources with the longitudinal change rate of hippocampus volume (N=2723)

		total			hippocampus(left)			hippocampus(right)		
		β	SE	P	β	SE	P	β	SE	P
Red meat	0	reference			reference			reference		
	lower intake	0.001289	0.001885	0.4944	0.001584	0.002794	0.571	0.001028	0.002504	0.681
	higher intake	0.003206	0.002171	0.1398	0.004333	0.003217	0.178	0.003177	0.002883	0.271
processed meat	0	reference			Reference			reference		
	lower intake	0.001934	0.001985	0.3298	0.002031	0.00294	0.490	0.002854	0.002636	0.279
	higher intake	-0.001909	0.002023	0.3452	-0.004362	0.002997	0.146	-0.000308	0.002686	0.909
poultry	0	reference			reference			reference		
	lower intake	0.001441	0.001834	0.4322	-0.0008925	0.002718	0.7427	0.003271	0.002435	0.179
	higher intake	0.001641	0.002389	0.4922	0.003167	0.00354	0.371	0.00224	0.003171	0.48
eggs	0	reference			reference			reference		
	lower intake	-0.002186	0.002069	0.2907	0.00005471	0.003068	0.986	-0.003451	0.002745	0.2088
	higher intake	0.002897	0.002182	0.1845	-0.0002213	0.003236	0.946	0.005598	0.002896	0.0534
cheese	0	reference			reference			reference		
	lower intake	0.003776	0.002095	0.0716	0.007097	0.003104	0.0223	0.00152	0.002784	0.585
	higher intake	0.002528	0.002121	0.2334	0.004368	0.003142	0.1646	0.001013	0.002818	0.719
nuts	0	reference			reference			reference		
	lower intake	0.004524	0.002004	0.0241	0.005238	0.00297	0.0778	0.004435	0.002663	0.096
	higher intake	-0.0009412	0.002097	0.6536	-0.004306	0.003107	0.166	0.002172	0.002787	0.436
yogurt	0	reference			reference			reference		
	lower intake	0.0008537	0.00202	0.6727	-0.0006831	0.002994	0.8195	0.002399	0.002682	0.371
	higher intake	0.0006205	0.002032	0.7601	0.00179	0.003011	0.5521	0.0002294	0.002698	0.932
whole grains	0	reference			reference			reference		
	lower intake	-0.001092	0.002405	0.6497	-0.001267	0.003562	0.722	-0.001545	0.003194	0.628
	higher intake	0.001324	0.002444	0.5881	0.003501	0.003621	0.3336	-0.000328	0.003246	0.92
legumes	0	reference			reference			reference		
	lower intake	0.0001805	0.001972	0.9271	3.061e-06	0.002923	0.9992	0.001017	0.002619	0.698

	higher intake	0.0005184	0.001974	0.7929	0.0006546	0.002925	0.8229	0.0007969	0.002621	0.761
milk	0	reference			reference			reference		
	lower intake	0.0004236	0.003044	0.8893	-0.003624	0.00451	0.4218	0.003652	0.004042	0.366
	higher intake	0.001149	0.003077	0.7089	-0.001162	0.004559	0.7989	0.002848	0.004085	0.486
sea food	0	reference			reference			reference		
	lower intake	0.00233	0.001966	0.2361	0.0004649	0.002916	0.8733	0.00468	0.002611	0.0732
	higher intake	0.004514	0.002095	0.0313	0.003269	0.003107	0.2929	0.005527	0.002782	0.047

Supplemental Table S6 Association between dietary protein intake with the longitudinal change rate of hippocampus volume in male(N=1337)

	hippocampus(left)			hippocampus(right)			hippocampus(total)		
	β	SE	P	β	SE	P	β	SE	P
total protein									
model1	-7.47E-05	7.934E-05	0.347	7.540E-05	6.83E-05	0.270	-1.47E-05	5.15E-05	0.776
model2	6.86E-05	1.173E-04	0.559	8.441E-05	1.01E-04	0.400	5.51E-05	7.61E-05	0.470
model3	6.55E-05	1.176E-04	0.577	8.303E-05	1.01E-04	0.413	5.33E-05	7.63E-05	0.485
animal/protein									
model1	0.0267957	1.719E-02	0.119	2.697E-02	1.480E-02	0.069	2.33E-02	1.13E-02	0.040
model2	2.625E-02	1.744E-02	0.132	2.628E-02	1.50E-02	0.081	2.37E-02	1.13E-02	0.036
model3	2.58E-02	1.749E-02	0.140	2.568E-02	1.51E-02	0.089	2.465E-02	1.115E-02	0.027
vegetable/protein									
model1	-2.680E-02	1.719E-02	0.119	-2.697E-02	1.480E-02	0.069	-2.465E-02	1.115E-02	0.027
model2	-2.625E-02	1.744E-02	0.132	-2.630E-02	1.50E-02	0.081	-2.37E-02	1.13E-02	0.036
model3	-2.581E-02	1.749E-02	0.140	-2.570E-02	1.51E-02	0.089	-2.33E-02	1.13E-02	0.040
vegetable protein									
model1	-5.298E-04	1.951E-04	0.007	7.880E-05	1.68E-04	0.640	-2.987E-04	0.0001267	0.019
model2	-5.399E-04	2.636E-04	0.0408	2.650E-04	2.28E-04	0.244	-3.66E-04	1.71E-04	0.033
model3	-5.381E-04	2.642E-04	0.0419	2.590E-04	2.28E-04	0.257	-3.63E-04	1.71E-04	0.035
animal protein									
model1	1.657E-05	9.127E-05	0.856	1.169E-04	7.85E-05	0.137	4.56E-05	5.92E-05	0.441
model2	1.507E-04	1.087E-04	0.166	1.18E-04	9.38E-05	0.210	1.10E-04	7.06E-05	0.121
model3	1.479E-04	1.090E-04	0.175	1.15E-04	9.40E-05	0.220	1.08E-04	7.07E-05	0.129
animal/vegetable									
model1	1.446E-02	8.428E-03	0.086	1.336E-02	7.254E-03	0.066	1.287E-02	5.465E-03	0.019
model2	1.390E-02	8.552E-03	0.104	1.300E-02	7.37E-03	0.078	1.226E-02	5.546E-03	0.027
model3	1.369E-02	8.575E-03	0.111	1.280E-02	7.39E-03	0.084	1.208E-02	5.560E-03	0.030

Supplemental Table S7 Association between dietary protein intake with the longitudinal change rate of hippocampus volume in female(N=1386)

	hippocampus(left)			hippocampus(right)			hippocampus(total)		
	β	SE	P	β	SE	P	β	SE	P
total protein									
model1	1.19E-04	7.105E-05	0.095	4.250E-05	6.75E-05	0.52914	3.44E-05	5.08E-05	0.4987
model2	1.37E-04	9.695E-05	0.159	4.44E-05	9.22E-05	0.62983	9.03E-05	6.93E-05	0.1924
model3	1.42E-04	9.716E-05	0.145	4.607E-05	9.21E-05	0.61682	9.22E-05	6.94E-05	0.184
animal/protein									
model1	2.467E-02	1.160E-02	0.034	0.0212083	0.011016	0.0544	0.0229959	0.0082765	0.00553
model2	2.433E-02	1.178E-02	0.039	2.35E-02	1.12E-02	0.03598	2.41E-02	8.40E-03	0.00422
model3	2.426E-02	1.180E-02	0.040	2.33E-02	1.12E-02	0.0373	2.40E-02	8.42E-03	0.00442
vegetable/protein									
model1	-2.467E-02	1.160E-02	0.034	-0.0212083	0.011016	0.0544	-0.0229958	0.0082765	0.005535
model2	-2.433E-02	1.178E-02	0.039	-2.35E-02	1.12E-02	0.03598	-2.41E-02	8.40E-03	0.00422
model3	-2.426E-02	1.180E-02	0.040	-2.33E-02	1.12E-02	0.0373	-2.40E-02	8.42E-03	0.00442
vegetable protein									
model1	-0.0001899	1.126E-04	0.092	-9.58E-05	1.58E-04	0.543494	-0.0002648	0.0001496	0.07693
model2	-2.67E-04	1.435E-04	0.063	-2.56E-04	2.01E-04	0.20283	-2.60E-04	1.91E-04	0.17373
model3	-2.64E-04	1.438E-04	0.067	-2.51E-04	2.02E-04	0.21351	-2.58E-04	1.91E-04	0.17657
animal protein									
model1	1.63E-04	7.725E-05	0.035	1.34E-05	7.35E-05	0.85516	8.64E-05	5.52E-05	0.118
model2	1.57E-04	8.677E-05	0.070	8.54E-05	8.24E-05	0.3004	1.22E-04	6.20E-05	0.0488
model3	1.60E-04	8.692E-05	0.066	8.37E-05	8.25E-05	0.31025	1.23E-04	6.21E-05	0.0478
animal/vegetable									
model1	1.186E-04	7.105E-05	0.095	4.25E-05	6.75E-05	0.52914	3.437E-05	5.080E-05	0.4987
model2	1.366E-04	9.695E-05	0.159	4.61E-05	9.21E-05	0.61682	9.034E-05	6.927E-05	0.1924
model3	1.415E-04	9.716E-05	0.145	4.44E-05	9.22E-05	0.62983	9.217E-05	6.941E-05	0.184

Supplemental Table S8 Association between dietary protein intake with the longitudinal change rate of hippocampus volume adjusted for AD-PRS(N=1386)

	hippocampus(left)			hippocampus(total)			hippocampus(right)		
	β	SE	P	β	SE	P	β	SE	P
total protein	9.31E-05	7.71E-05	0.2273	6.59E-05	5.19E-05	0.2046	6.21E-05	6.88E-05	0.36724
animal/protein	2.53E-02	1.04E-02	0.0149	2.36E-02	6.97E-03	0.000724	2.40E-02	9.25E-03	0.00954
vegetable/protein	-2.36E-02	6.97E-03	0.000724	-2.53E-02	1.04E-02	0.0149	-2.40E-02	9.25E-03	0.00954
vegetable protein	-3.06E-04	1.12E-04	0.00644	-3.91E-04	1.67E-04	0.0195	-2.50E-04	1.49E-04	0.09411
animal protein	1.10E-04	4.75E-05	0.0209	1.48E-04	7.05E-05	0.0365	9.64E-05	6.30E-05	0.12566
animal/vegetable	1.13E-02	3.44E-03	0.000976	1.24E-02	5.11E-03	0.015	1.13E-02	4.56E-03	0.01356

The analyses were based on Model 3 in the main analysis (adjusted for age, sex, ethnicity, Townsend deprivation index, education level, physical activity, smoking, body weight status, total energy intake, baseline cancer, CVDs, hypertension, and diabetes) with additional adjustment for polygenic risk scores of Alzheimer's diseases (AD-PRS).

Supplemental Table S9 Association between dietary protein intake with the longitudinal change rate of hippocampus volume among participants with at least two waves of dietary data (N=2009)

	hippocampus(left)			hippocampus(total)			hippocampus(right)		
	β	SE	P	β	SE	P	β	SE	P
total protein	1.88E-04	1.03E-04	0.067	1.18E-04	6.92E-05	0.089	8.67E-05	9.15E-05	0.344
animal/protein	3.00E-02	1.29E-02	0.020	2.80E-02	8.69E-03	0.001	2.91E-02	1.15E-02	0.011
vegetable/protein	-3.00E-02	1.29E-02	0.020	-2.80E-02	8.69E-03	0.001	-2.91E-02	1.15E-02	0.011
vegetable protein	-4.51E-04	2.09E-04	0.031	-3.81E-04	1.41E-04	0.007	-3.57E-04	1.87E-04	0.056
animal protein	2.37E-04	9.17E-05	0.010	1.67E-04	6.18E-05	0.007	1.38E-04	8.18E-05	0.092
animal/vegetable	1.42E-02	6.38E-03	0.026	1.32E-02	4.30E-03	0.002	1.37E-02	5.68E-03	0.016

The model was adjusted for age, sex, ethnicity, Townsend deprivation index, education level, physical activity, smoking, body weight status, total energy intake, baseline cancer, CVDs, hypertension, and diabetes among participants with at least two waves of dietary data.

Supplemental Table S10 Association between dietary protein intake with the longitudinal change rate of hippocampus volume with excluding baseline neuropsychiatric disorders (N=2398)

	hippocampus(left)			hippocampus(total)			hippocampus(right)		
	β	SE	P	β	SE	P	β	SE	P
total protein	9.466E-05	8.092E-05	0.242	5.457E-05	5.276E-05	0.301	7.757E-05	6.838E-05	0.257
animal/protein	2.399E-02	1.125E-02	0.038	1.899E-02	7.329E-03	0.010	2.552E-02	9.277E-03	0.001
vegetable/protein	-2.339E-02	1.125E-02	0.038	-1.899E-02	7.329E-03	0.010	-2.552E-02	9.277E-03	0.001
vegetable protein	-3.701E-04	1.821E-04	0.042	-2.394E-04	1.188E-04	0.044	-2.651E-04	1.508E-04	0.001
animal protein	1.407E-04	7.410E-05	0.050	8.542E-05	4.832E-05	0.077	1.107E-04	6.258E-05	0.077
animal/vegetable	1.169E-02	5.564E-03	0.036	9.216E-03	3.626E-03	0.011	-1.222E-02	4.607E-03	0.008

The analyses were based on Model 3 in the main analysis (adjusted for age, sex, ethnicity, Townsend deprivation index, education level, physical activity, smoking, body weight status, total energy intake, baseline cancer, CVDs, hypertension, and diabetes) with excluding baseline neuropsychiatric disorders.

Supplemental Table S11 Association between dietary protein intake with the longitudinal change

rate of hippocampus volume in female adjusted for oral contraceptive (N=1386)

	hippocampus(left)			hippocampus(total)			hippocampus(right)		
	β	SE	P	β	SE	P	β	SE	P
total protein	9.779E-05	7.585E-05	0.197	7.617E-05	5.118E-05	0.137	7.763E-05	6.799E-05	0.254
animal/protein	2.509E-02	1.021E-02	0.014	2.443E-02	6.885E-03	0.001	2.570E-02	9.153E-03	0.005
vegetable/protein	-2.509E-02	1.021E-02	0.014	-2.443E-02	6.885E-03	0.001	-2.570E-02	9.153E-03	0.005
vegetable protein	-3.906E-04	1.648E-04	0.018	-3.179E-04	1.112E-04	0.004	-2.703E-04	1.478E-04	0.068
animal protein	1.506E-04	6.925E-05	0.030	1.197E-04	4.672E-05	0.011	1.125E-04	6.209E-05	0.070
animal/vegetable	1.243E-02	5.033E-03	0.014	1.176E-02	3.393E-03	0.001	1.204E-02	4.510E-03	0.008

The analyses were based on Model 3 in the main analysis (adjusted for age, sex, ethnicity, Townsend deprivation index, education level, physical activity, smoking, body weight status, total energy intake, baseline cancer, CVDs, hypertension, and diabetes) with additional adjustment for oral contraceptive.
